# Baseline and longitudinal patterns of Non-HDL-C to HDL-C ratio (NHHR) and major adverse cardiovascular events in peripheral artery disease

**DOI:** 10.1038/s41598-025-19856-0

**Published:** 2025-10-15

**Authors:** Yujia Zhang, Zhoudong Jing, Zhiqiang Liu, Angel Lai, GuangMing Tan, Bryan P. Yan

**Affiliations:** Division of Cardiology, Department of Medicine and Therapeutics, Faculty of Medicine, The Chinese University of Hong Kong, Shatin, NT, Hong Kong China

**Keywords:** NHHR, PAD, Cardiovascular adverse events, CDARS, Biomarkers, Cardiology, Diseases, Medical research, Risk factors

## Abstract

The non-high-density lipoprotein cholesterol to high-density lipoprotein cholesterol ratio (NHHR) is an innovative lipid parameter for atherosclerosis risk, yet its association with major adverse cardiovascular events (MACE) in peripheral artery disease (PAD) patients remains unclear. We retrospectively analyzed 13,224 PAD patients in Hong Kong, assessing baseline NHHR and longitudinal NHHR trajectories as predictors of MACE using spline models, latent class growth modeling, and Cox proportional hazards models. Over a median follow-up of 3.4 years, a U-shaped relationship emerged between baseline NHHR and MACE risk. Patients in the highest quintile of NHHR (Q5) had a significantly increased risk of MACE (HR = 1.16, 95% CI: 1.07–1.25, *P* < 0.001), as did those in the lowest quintile (Q1) (HR = 1.17, 95% CI: 1.08–1.27, *P* < 0.001), compared with the reference group. Among five NHHR trajectories, moderate-high-stable (HR = 1.17 [95% CI: 1.04–1.32], *P* < 0.001) and high-stable groups (HR = 1.35 [95% CI: 1.14–1.60], *P* < 0.001) showed higher risk, whereas the declining group had no increased risk (HR = 1.04 [95% CI: 0.88–1.23], *P* = 0.356). High and low baseline NHHR, as well as persistently elevated trajectories, were associated with higher MACE risk in PAD, whereas declining trajectories normalized this risk. NHHR may serve as a useful marker for cardiovascular risk stratification in PAD patients.

**Supplementary Information:**

The online version contains supplementary material available at 10.1038/s41598-025-19856-0.

## Introduction

Peripheral artery disease (PAD) is the third most prevalent cardiovascular condition, following coronary artery disease (CAD) and stroke^1^. Globally, PAD affects approximately 230 million individuals, with its incidence increasing due to aging populations^2,3^. Patients with PAD are at high risk of cardiovascular and limb-related complications^4,5^, with cardiovascular events being the leading cause of mortality^6^. Therefore, effective management of cardiovascular risk factors is essential to reducing adverse outcomes in PAD patients. Among these, dyslipidemia has been recognized as a major modifiable risk factor contributing to the progression of atherosclerosis^7^.

Non-High-Density Lipoprotein Cholesterol (Non-HDL-C) includes the cholesterol content of all atherogenic lipoproteins. Since it is easily obtainable and unaffected by fasting status or triglyceride variations, it has gained increasing recognition as a reliable lipid parameter in recent years^8,9^. A study has demonstrated that non-HDL-C is a more effective predictor of cardiovascular events compared to low-density lipoprotein cholesterol (LDL-C) alone^10^, and the American College of Cardiology (ACC) guidelines recommended non-HDL-C as a treatment target for all CAD risk groups in 2017^11^. Building on the concept of non-HDL-C, the non-HDL-C to high-density lipoprotein cholesterol (HDL-C) ratio (NHHR) has gained attention as a potential biomarker for atherosclerotic risk assessment. Since NHHR includes both atherogenic and protective lipoprotein components^12^, it provides a broader perspective on lipid dynamic balance and its association with disease. Several studies have reported that NHHR provides superior predictive value compared with individual lipid markers like LDL-C and non-HDL-C in predicting coronary heart disease (CHD) risk^13,14^. Moreover, NHHR has also shown clinical significance in stratifying susceptibility to various diseases, including diabetes, abdominal aortic aneurysm, and metabolic syndrome^12,15–17^. However, the prognostic significance of NHHR in PAD patients remains insufficiently understood. This study uses the Clinical Data Analysis and Reporting System (CDARS) database to explore the association between NHHR and major adverse cardiovascular events (MACE) in patients with PAD, aiming to inform clinical lipid management strategies and improve patient prognosis.

## Materials and methods

This study received approval from the Institutional Review Board of The Chinese University of Hong Kong and the New Territories East Cluster under the Hospital Authority (Joint CUHK-NTEC CREC, Ref No. 2014.653). All methods were performed in accordance with relevant guidelines and regulations, including the Declaration of Helsinki. Due to the use of fully anonymized registry data, the requirement for written informed consent was waived.

Data Source.

This study used anonymized medical records from the CDARS, a comprehensive territory-wide electronic health record repository managed by Hong Kong’s Hospital Authority (HA). CDARS includes extensive clinical data from all public hospitals and clinics under HA management, covering demographic characteristics, vital status indicators, disease diagnoses, surgical procedures, medication records, and continuous laboratory test results^18^. The database covers approximately 80% of Hong Kong’s population, which totals 7.4 million.

Study design and population.

PAD patients were identified using the International Classification of Diseases, 9th Revision, Clinical Modification (ICD-9-CM) codes, with confirmed diagnostic encounters between July 15, 2003, and December 31, 2023^1^. Individuals were excluded based on the following criteria: age ≤ 18 years (*n* = 29), missing baseline NHHR data (*n* = 10,727), or MACE within 90 days post-PAD diagnosis (*n* = 3,661) to mitigate reverse causation bias by ensuring temporal sequence validity between exposure and outcome. After applying these criteria, 13,224 eligible patients were included in the analytical cohort (Additional File Figure S1). The baseline date was defined as the first recorded date of PAD diagnosis. Follow-up continued until the occurrence of MACE, death, the last follow-up date (December 31, 2023), or a maximum follow-up period of 5 years, whichever occurred first.

Data collection and definition.

Baseline characteristics included age, sex, comorbidities, laboratory parameters, and medication use. Comorbidities were identified based on diagnoses recorded before the PAD diagnosis, with each condition defined using its corresponding ICD-9-CM (Additional File Table S1). NHHR was calculated using non-HDL-C and HDL-C, with non-HDL-C derived by subtracting HDL-C from total cholesterol (TC).

Study Endpoints.

The primary endpoint was the occurrence of MACE, which included all-cause mortality, non-fatal myocardial infarction(non-fatal MI), and non-fatal stroke(ischemic stroke only)^19^. Mortality information was obtained from CDARS. Non-fatal MI was identified using ICD-9-CM code 410, and non-fatal ischemic stroke was defined using ICD-9-CM codes 433 and 434, with explicit exclusion of hemorrhagic stroke and non-acute cerebrovascular conditions.

### Statistical analysis

Baseline characteristics across NHHR quintiles (Q1–Q5, from lowest to highest) were compared using the chi-square test or Fisher’s exact test for categorical variables, and analysis of variance (ANOVA) or the Kruskal-Wallis test for continuous variables, depending on the data distribution and homogeneity of variance. Continuous variables were reported as means with standard deviations (SD) or medians with interquartile ranges (IQR), as appropriate, while categorical variables were expressed as frequencies with percentages.

Missing data were handled using multivariate imputation by chained equations (MICE) based on random forests to minimize imputation bias, which can otherwise lead to reduced statistical power and biased estimates.

Kaplan-Meier survival curves were generated to assess the incidence of MACE across the five NHHR categories. Cox proportional hazards models were used to calculate hazard ratios (HRs) and their 95% confidence intervals (CIs) for NHHR. Patients in the third quintile (Q3) of baseline NHHR were designated as the reference group. Both unadjusted and adjusted HRs were estimated, with adjustments for age, sex, history of hypertension, diabetes mellitus (DM), CAD, stroke, estimated glomerular filtration rate (eGFR), hemoglobin A1c (HbA1c), and medication use (antiplatelet agents, angiotensin-converting enzyme inhibitors/angiotensin II receptor blockers [ACEI/ARBs], and statins). The selection of covariates was guided by prior studies^20,21^ and clinical relevance. Variance inflation factors (VIFs) for all covariates were < 2, indicating no significant collinearity (Additional file Table S2). The proportional hazards assumption was tested by incorporating a time-dependent interaction term between NHHR categories and follow-up time.

To examine potential non-linear associations, we used spline functions in conjunction with the R package “smoothHR”^22^, allowing for flexible modeling of NHHR and their relationship with MACE. The HRs curves were adjusted for the aforementioned covariates, with the third quintile (Q3) of NHHR group treated as the reference group. A continuous spectrum of NHHR was employed to compute the 95% CIs.

We further explored whether the relationship between NHHR and MACE varied across sex (male/female), age (< 65 years/≥65 years), DM (yes/no), and hypertension (yes/no) through interaction analyses, with adjustments for the aforementioned covariates.

Additionally, to evaluate the impact of long-term NHHR patterns on MACE, we applied a latent class growth model (LCGM) to 6,557 PAD patients with at least four NHHR measurements. To ensure valid trajectory assignment, patients were required to have all NHHR measurements taken before any outcome event, a measurement interval ≥ 6 months, and no measurements within 3 months prior to the outcome to reduce short-term fluctuations. Several LCGM specifications, ranging from 3 to 7 latent groups with varying polynomial degrees, were tested to determine the optimal model. The determination of the final trajectory groups was guided by clinical relevance and the following criteria: (1) the lowest Bayesian information criterion (BIC) value^23^; (2) an average posterior probability exceeding 0.7 for all latent classes^24^; and (3) no fewer than 5% of participants in any group. For outcome analyses, group membership was treated as an independent variable in Cox regression. All covariates were defined at baseline to ensure comparability across groups, minimize potential bias from covariates influenced by prior NHHR exposure.

The robustness of the findings was evaluated through multiple sensitivity analyses. First, to reduce potential bias, participants who encountered MACE during the initial year of follow-up were excluded. Second, participants with a prior history of stroke or MI were excluded to mitigate the potential impact of past cardiovascular events on recurrence risk. Third, patients with baseline neoplastic diseases, hematologic disorders, and advanced renal or hepatic insufficiency were excluded to limit competing risks. Fourth, patients with MACE within 90 days of diagnosis were included to assess the robustness of the results. Fifth, we re-evaluated the association using Cox proportional hazards models with varying sets of covariate adjustments. Finally, in the trajectory analysis, patients whose time interval between the first and last NHHR measurements was less than one year were omitted to further enhance the reliability of our findings.

## Results

### Characteristics according to NHHR categories

Baseline clinical characteristics were recorded for 13,224 patients, with a median age of 74 (65–82) years. The cohort was composed of 60.8% males. Table 1 summarizes the characteristics stratified by baseline NHHR categories. The distribution of baseline NHHR values is shown in Additional File Figure S2. Notably, patients with higher baseline NHHR were generally younger, predominantly male, and showed higher levels of TC, triglycerides (TG), LDL-C, and HbA1c, along with lower HDL-C. To assess potential selection bias, we compared baseline characteristics between patients with versus without baseline NHHR and between those with ≥ 4 versus < 4 measurements. Patients without baseline NHHR were older with lower medication use, while other clinical characteristics were broadly comparable; patients with ≥ 4 measurements were younger, but their comorbidity burden and baseline lipid levels were similar (Additional File Tables S3–S4).

**Table 1:** Baseline characteristics of PAD patients stratified by NHHR categories. Values are presented as mean ± SD, median (IQR), or n (%), as appropriate. NHHR non-high-density lipoprotein cholesterol to high-density lipoprotein cholesterol ratio, CAD coronary artery disease, CKD chronic kidney disease, TC total cholesterol, LDL-C low-density lipoprotein cholesterol, HDL-C high-density lipoprotein cholesterol, TG triglycerides, eGFR estimated glomerular filtration rate, HbA1c glycated hemoglobin, ACEI angiotensin-converting enzyme inhibitor, ARBs angiotensin receptor blockers.

Parameters	Total *N* = 13,224	NHHR(Q1) *N* = 2,645	NHHR(Q2) *N* = 2,645	NHHR(Q3) *N* = 2,652	NHHR(Q4) *N* = 2,638	NHHR(Q5) *N* = 2,644	*P* value
NHHR	2.68 (1.93, 3.66)	1.45 (1.19, 1.63)	2.08 (1.93, 2.23)	2.68 (2.53, 2.84)	3.42 (3.20, 3.66)	4.81 (4.31, 5.63)	< 0.001
Demographic							
Age, years	74.00 (65.00, 82.00)	77.00 (68.00, 84.00)	75.00 (67.00, 83.00)	74.50 (65.00, 82.00)	73.00 (64.00, 81.00)	70.00 (61.00, 79.00)	< 0.001
Sex (Male), %	8035 (60.8%)	1470 (55.6%)	1561 (59.0%)	1626 (61.3%)	1662 (63.0%)	1716 (64.9%)	< 0.001
Comorbidities							
Hypertension, %	7700 (58.2%)	1616 (61.1%)	1610 (60.9%)	1538 (58.0%)	1474 (55.9%)	1462 (55.3%)	< 0.001
CAD, %	3927 (29.7%)	882 (33.3%)	854 (32.3%)	754 (28.4%)	726 (27.5%)	711 (26.9%)	< 0.001
Stroke, %	3021 (22.8%)	717 (27.1%)	625 (23.6%)	596 (22.5%)	566 (21.5%)	517 (19.6%)	< 0.001
Dyslipidemia, %	876 (6.6%)	225 (8.5%)	179 (6.8%)	180 (6.8%)	162 (6.1%)	130 (4.9%)	< 0.001
CKD, %	2896 (21.9%)	498 (18.8%)	541 (20.5%)	532 (20.1%)	597 (22.6%)	728 (27.5%)	< 0.001
Diabetes mellitus, %	7160 (54.1%)	1327 (50.2%)	1404 (53.1%)	1430 (53.9%)	1467 (55.6%)	1532 (57.9%)	< 0.001
Laboratory							
TC, mmol/L	4.22 (3.58, 5.00)	3.70 (3.11, 4.30)	3.90 (3.33, 4.50)	4.16 (3.60, 4.80)	4.50 (3.87, 5.20)	5.20 (4.43, 6.04)	< 0.001
LDL-C, mmol/L	2.37(1.81,3.06)	1.72 (1.35, 2.12)	2.11 (1.73, 2.57)	2.41 (1.97, 2.93)	2.73 (2.21, 3.30)	3.30 (2.62, 4.00)	< 0.001
HDL-C, mmol/L	1.13 (0.93, 1.39)	1.55 (1.30, 1.83)	1.27 (1.09, 1.47)	1.13 (0.99, 1.30)	1.01 (0.87, 1.18)	0.86 (0.73, 1.00)	< 0.001
TG, mmol/L	1.25 (0.91, 1.76)	0.87 (0.69, 1.10)	1.06 (0.84, 1.36)	1.25 (0.98, 1.62)	1.48 (1.12, 1.97)	1.98 (1.46, 2.76)	< 0.001
eGFR, mL/min/1.73 m²	63.71 (40.24, 85.60)	65.07 (43.33, 85.70)	64.93 (41.74, 85.63)	65.45 (43.20, 86.74)	63.67 (40.24, 84.55)	57.95 (32.16, 84.73)	< 0.001
HbA1c, %	7.10 (6.20, 8.48)	6.70 (5.92, 7.63)	6.90 (6.10, 8.00)	7.12 (6.30, 8.40)	7.32 (6.31, 8.89)	7.70 (6.50, 9.50)	< 0.001
Medication							
Anticoagulant, %	1073 (8.1%)	288 (10.9%)	240 (9.1%)	206 (7.8%)	187 (7.1%)	152 (5.7%)	< 0.001
ACEI/ARBs, %	7169 (54.2%)	1504 (56.9%)	1481 (56.0%)	1448 (54.6%)	1410 (53.4%)	1326 (50.2%)	< 0.001
β-blockers, %	5093 (38.5%)	958 (36.2%)	1037 (39.2%)	1031 (38.9%)	1022 (38.7%)	1045 (39.5%)	0.1008
Statin, %	7675 (58.0%)	1775 (67.1%)	1662 (62.8%)	1514 (57.1%)	1408 (53.4%)	1316 (49.8%)	< 0.001
Antiplatelet, %	9485 (71.7%)	1955 (73.9%)	1945 (73.5%)	1933 (72.9%)	1844 (69.9%)	1808 (68.4%)	< 0.001
Antihyperglycemic treatment, %	4058 (30.7%)	739 (27.9%)	756 (28.6%)	790 (29.8%)	844 (32.0%)	929 (35.1%)	< 0.001

### Association between baseline NHHR and the risk of MACE

**Fig. 1:**
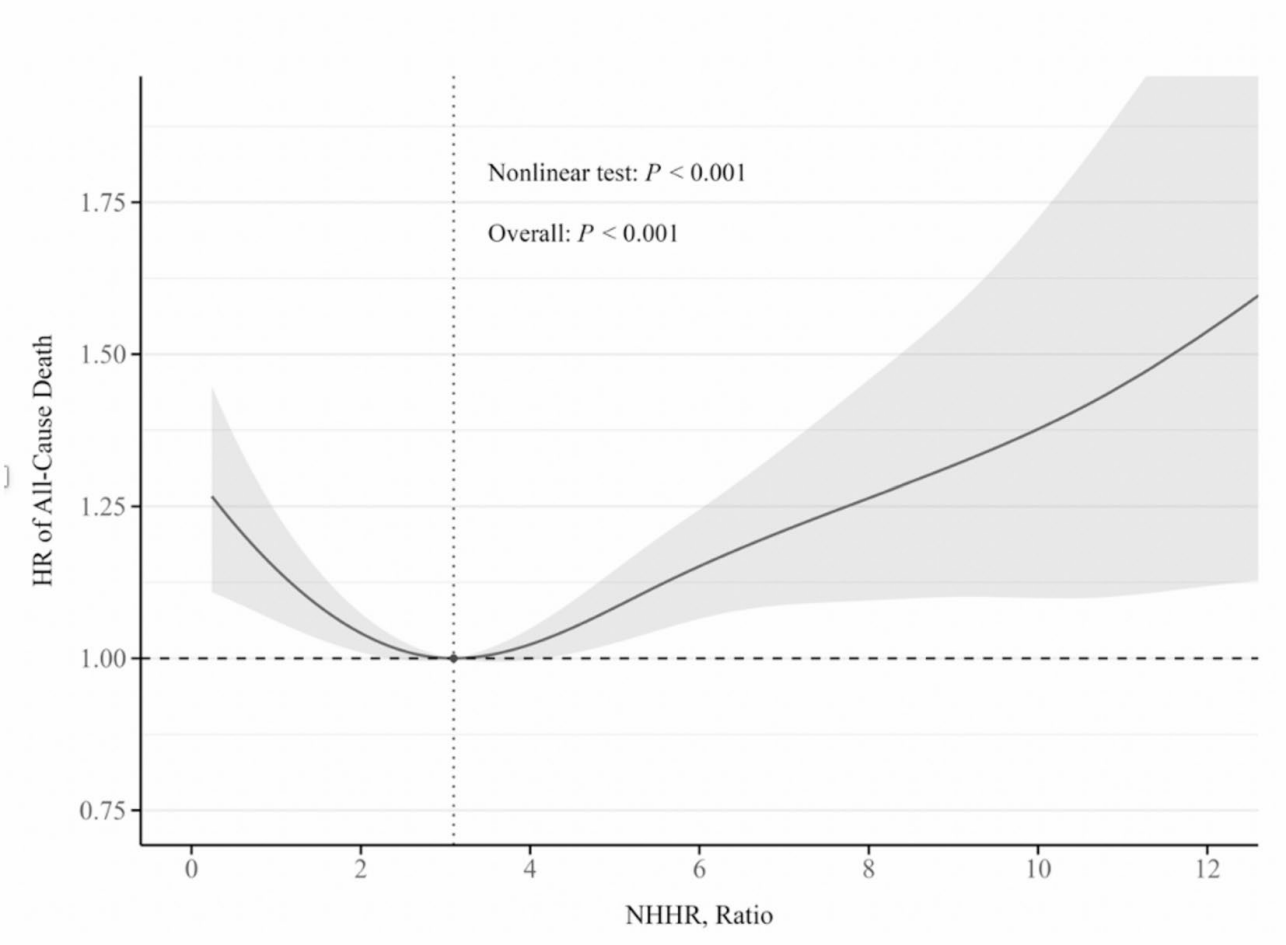
Nonlinear association between baseline NHHR and MACE. There is a U-shaped association between baseline NHHR and the hazard ratio of MACE. HR is represented by solid line, with 95% CIs shown as shaded areas. Model was adjusted for sex, age, hypertension, DM, CAD, stroke, HbA1c, eGFR, and the use of antiplatelets, ACEI/ARBs, and statins. HR hazard ratio, CI confidence interval, DM diabetes mellitus, CAD coronary artery disease, HbA1c glycated hemoglobin, eGFR estimated glomerular filtration rate, ACEI angiotensin-converting enzyme inhibitor, ARBs angiotensin receptor blockers.

**Fig. 2:**
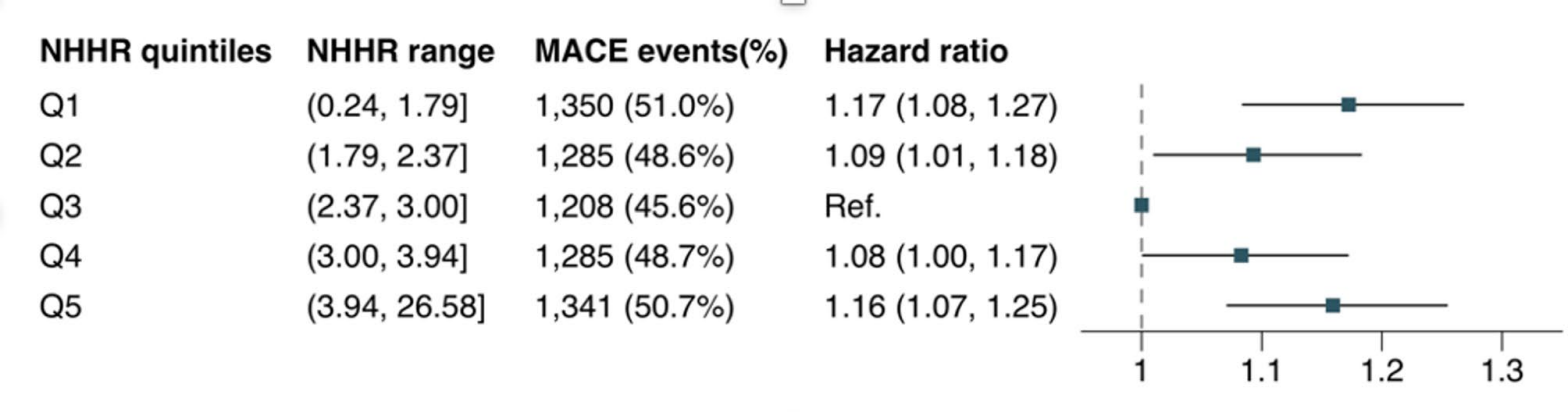
Adjusted HRs for MACE across baseline NHHR categories. NHHR was categorized into quintiles (Q1–Q5), with NHHR levels increasing across groups; Q3 was used as the reference group. Compared with Q3, the risk of MACE was significantly higher in Q1 (HR = 1.17 [95%Cl: 1.08–1.27], *P* < 0.001) and Q5 (HR = 1.16 [95%Cl: 1.07–1.25], *P* < 0.001). Elevated risks were also observed in Q2 (HR = 1.09 [95%Cls: 1.01–1.18], *P* = 0.027) and Q4 (HR = 1.08 [95%Cls: 1.00–1.17], *P* = 0.047). Model was adjusted for sex, age, hypertension, DM, CAD, stroke, HbA1c, eGFR, and the use of antiplatelets, ACEI/ARBs, statins. HR hazard ratio, CI confidence interval, DM diabetes mellitus, CAD coronary artery disease, HbA1c glycated hemoglobin, eGFR estimated glomerular filtration rate, ACEI angiotensin-converting enzyme inhibitor, ARBs angiotensin receptor blockers.

Over a median follow-up of 3.4 years, 6,469 (48.9%) participants experienced MACE, which included 4,860 (75.1%) all-cause death cases, 1,017 (15.7%) non-fatal MI cases, and 592 (9.2%) non-fatal stroke cases. The event rates of MACE across NHHR categories are presented in Additional File Figure S3. Kaplan-Meier analysis demonstrated a non-monotonic relationship between baseline NHHR categories and MACE incidence (log-rank *P* < 0.001), with the lowest cumulative risk observed in the third NHHR(Q3) group (Additional File Figure S4). This group was selected as the reference for subsequent analyses. A U-shaped association between baseline NHHR and MACE risk was confirmed after multivariable adjustment (P for nonlinearity < 0.001; Fig. 1). Patients in the lowest NHHR group(Q1) and the highest NHHR group (Q5) had significantly higher risks of MACE compared to those in the Q3 NHHR group in both unadjusted and adjusted models (Unadjusted: HR = 1.21 [95% Cl: 1.12–1.31], *P* < 0.001; HR = 1.15 [95%Cl: 1.07–1.25], *P* < 0.01, respectively; Adjusted: HR = 1.17 [95%Cl: 1.08–1.27], *P* < 0.001; HR = 1.16 [95%Cl: 1.07–1.25], *P* < 0.001, respectively). Furthermore, patients in the Q2 NHHR and Q4 NHHR groups also exhibited a significant increased risk of MACE after adjustment (HR = 1.09 [95%Cls: 1.01–1.18], *P* = 0.027; HR = 1.08 [95%Cls: 1.00–1.17], *P* = 0.047, respectively) (Fig. 2 and Additional File Table S5). Additionally, incremental analyses showed that NHHR significantly improved model fit (Likelihood Ratio Test *P* < 0.001) with consistent gains in discrimination and reclassification (Additional File Table S6). We also analyzed each component of MACE separately. The U-shaped association of NHHR was primarily observed for all-cause mortality. For non-fatal MI, only the highest quintile showed a significantly elevated risk compared with the reference group, and no significant associations were observed for non-fatal ischemic stroke (Additional File Figure S5-S7; Table S7-S9).

### Subgroup analysis

**Fig. 3:**
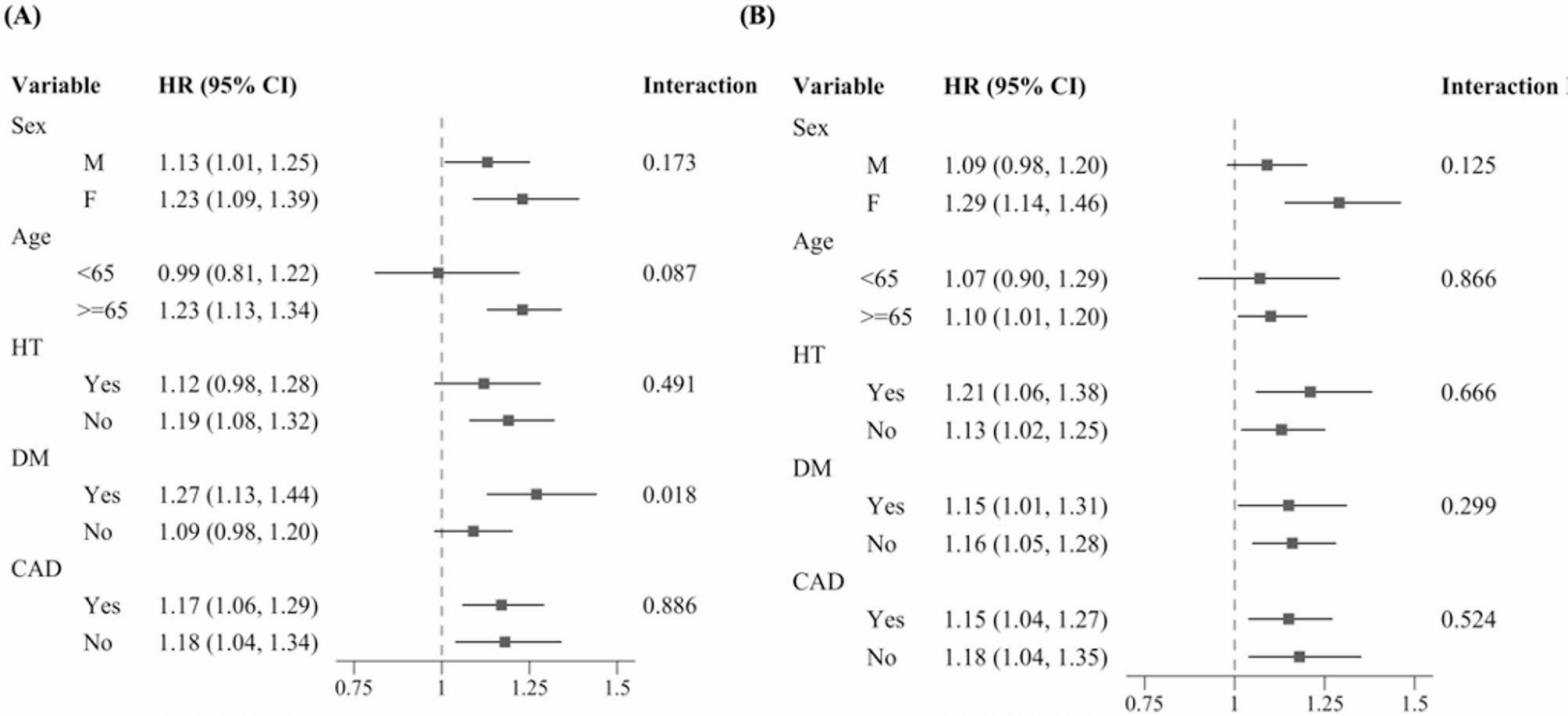
Subgroup analysis of the association between baseline NHHR and the occurrence of MACE (**A**) shows results for the Q1 NHHR group, where MACE risk was higher in patients with DM (HR = 1.27 [95% CI: 1.13–1.44], *P* < 0.001) than in those without DM (HR = 1.09 [95% CI: 0.98–1.20], *P* = 0.122).  Figure 3(**B**) shows results for the Q5 NHHR group, with Q3 NHHR serving as the reference group.

The NHHR Q1 (lowest) group demonstrated a significant interaction with DM for MACE (β = −0.172, SE = 0.079, *P* = 0.018; Fig. 3A). Patients with DM and low NHHR were at a higher risk of MACE compared to those without DM (HR = 1.27 [95%Cl: 1.13–1.44], *P* < 0.001 vs. HR = 1.09 [95%Cl: 0.98–1.20], *P* = 0.122). No other subgroup showed a significant interaction (Fig. 3A-B; Additional Table S10).

### Association between NHHR trajectory and MACE risk

**Fig. 4:**
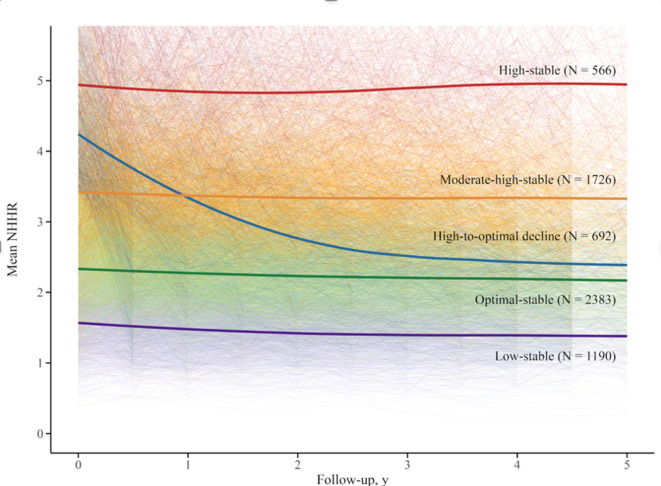
NHHR trajectories over the follow-up period were identified by LCGM. Five NHHR trajectories were identified: low-stable (*n* = 1,190), optimal-stable (*n* = 2,383; reference), moderate-high-stable (*n* = 1,726), high-stable (*n* = 566), and high-to-optimal decline (*n* = 692).

A total of 6,557 participants, each with at least four NHHR measurements, were included in trajectory analysis. Among them, 1,884 (28.73%) experienced MACE. Five distinct NHHR trajectories were identified from the time of PAD diagnosis until the occurrence of outcomes (Fig. 4). The average posterior probabilities for each latent trajectory class, ranging from 0.915 to 0.932, confirmed the robustness of the trajectory classification (Additional File Table S11). Four of these trajectories exhibited stable trends: 18% (*n* = 1,190, mean NHHR range: 1.35–1.59) maintained consistently low NHHR throughout the follow-up period (low-stable group); 36% (*n* = 2,383, mean NHHR range: 2.05–2.36) exhibited the second-highest NHHR levels, designated as the optimal-stable group, which served as the reference group; 26% (*n* = 1,726, mean NHHR range: 3.15–3.44) displayed moderate high NHHR levels (moderate high-stable group); and 9% (*n* = 566, mean NHHR range: 4.34–5.05) maintained the highest NHHR throughout the follow-up period (high-stable group). In addition, 11% (*n* = 692) of participants exhibited a mean NHHR decrease from 4.53 to 2.22, representing a trajectory of rapid and continuous NHHR reduction (high-to-optimal decline group).

**Fig. 5:**
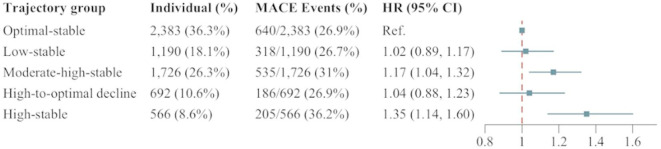
Associations of NHHR trajectories with the risk of MACE. Compared to the optimal-stable group, MACE risk was higher in the moderate-high-stable group (HR = 1.17, 95% CI: 1.04–1.32, *P* < 0.001) and the high-stable group (HR = 1.35, 95% CI: 1.14–1.60, *P* < 0.001). The high-to-optimal decline group had similar risk (HR = 1.04, 95% CI: 0.88–1.23, *P* = 0.356) to the optimal-stable group. Model was adjusted for sex, age, hypertension, DM, CAD, stroke, HbA1c, eGFR, and the use of antiplatelets, ACEI/ARBs, statins. HR hazard ratio, CI confidence interval, DM diabetes mellitus, CAD coronary artery disease, HbA1c glycated hemoglobin, eGFR estimated glomerular filtration rate, ACEI angiotensin-converting enzyme inhibitor, ARBs angiotensin receptor blockers.

Among the four groups with relatively stable NHHR, the reference group (optimal-stable group) MACE incidence was 26.9%, while the high-stable group MACE incidence was 36.2%. Compared to the optimal-stable group, both the moderate-high-stable group (HR = 1.17 [95%Cl: 1.04–1.32], *P* < 0.001) and the high-stable group (HR = 1.35 [95%Cl: 1.14–1.60], *P* < 0.001) demonstrated a notably increased MACE risk. Conversely, the high-to-optimal decline group showed a MACE risk comparable to that of the optimal-stable group, with no statistically significant difference observed (HR = 1.04 [95%Cl: 0.88–1.23], *P* = 0.356) (Fig. 5).

Sensitivity analyses consistently supported the robustness of our findings. These included exclusion of patients with events within the first year, exclusion of patients with a history of stroke or myocardial infarction, exclusion of individuals with cancer, hematologic disorders, and advanced liver or kidney disease, inclusion of patients who experienced MACE within 90 days, and analysis with different sets of covariate adjustments. Additionally, the robustness of longitudinal NHHR trajectories was confirmed by excluding patients with measurement intervals shorter than one year (Additional File Tables S12–S16 and Figures S8–S9).

## Discussion

Our study examines the association between NHHR and MaCE in PAD patients, incorporating both baseline NHHR and its long-term trajectories. We found a U-shape association between baseline NHHR and MACE risk, with the lowest risk observed among patients with NHHR values between 2 and 3. Consistently, trajectory analysis identified the group with mean NHHR levels between 2.05 and 2.36 that exhibited the most favorable prognosis. Together, these results converge on a similar range, suggesting that maintaining NHHR within a moderate range may be associated with reduced MACE risk. These findings suggest the potential of NHHR as a clinically useful marker for cardiovascular risk stratification and may help guide lipid management strategies in patients with PAD.

As previously mentioned, research has demonstrated a significant association between NHHR and atherosclerosis, outperforming traditional lipid markers in its prediction. However, to our knowledge, few studies have discussed the NHHR’s role in predicting MACE. One study reported that elevated NHHR correlated with a higher occurrence of MACE in CAD patients, but did not adjust for potential confounders or quantify the optimal NHHR range^25^. Another study investigated a U-shaped relationship between NHHR and MACE in CAD patients. Yet, it primarily focused on baseline NHHR while neglecting long-term patterns in NHHR and their contribution to MACE development^26^. PAD shares similar pathophysiological mechanisms with CAD, as both are manifestations of systemic atherosclerosis. However, lipid management and risk stratification in PAD receive less attention than in CAD. This gap highlights the relevance of our study, which investigates NHHR as a predictor of MACE in PAD patients. Indeed, recent evidence further suggests the use of lipid-related ratios as predictive markers in PAD. Evsen et al. demonstrated that the LDL/HDL ratio was superior to the plasma atherogenic index (AIP) in predicting PAD complexity^27^. Our findings are consistent with those of Liu et al., who examined the association between NHHR and MACCEs in a PCI population^26^.

We found that both high and low baseline NHHR are associated with a higher MACE risk, potentially through multiple underlying mechanisms. Excessively high NHHR: This condition is primarily driven by elevated non-HDL-C, which reflects the cholesterol content of all apoB-containing lipoproteins. These particles undergo subendothelial retention and oxidative modification, triggering vascular inflammation and endothelial dysfunction, while downstream processes such as cholesterol-crystal-induced inflammasome activation further enhance plaque vulnerability^28–30^. LDL-C, the major component of non–HDL-C, remains a well-established causal driver^28,31^. Small dense LDL is particularly atherogenic, and triglyceride-rich remnants and IDL contribute to residual risk through slow clearance and high cholesterol content^29,32^. Lipoprotein(a) further augments vascular injury and thrombosis via oxidized phospholipids and impaired fibrinolysis^33,34^. Low HDL-C, often accompanying high NHHR, further reduces cholesterol efflux and anti-inflammatory protection, thereby aggravating atherosclerosis^35,36^. Excessively low NHHR: It may result from disproportionately high HDL-C or very low non-HDL-C levels. The paradoxical risk associated with very high HDL-C is most plausibly explained by impaired functionality rather than concentration, as efflux capacity and anti-inflammatory activity are stronger predictors of cardiovascular protection than HDL-C levels themselves^37^. Under oxidative stress or systemic inflammation, HDL-C can lose its protective properties and even become pro-inflammatory^38,39^. Genetic variants in CETP, ABCA1, LIPC, and SCARB1 further highlight that elevated HDL-C levels do not necessarily confer benefit, as some variants paradoxically increase coronary risk^38,40,41^. In addition, the marked heterogeneity of HDL particles—in terms of size, composition, and function—may yield subfractions with diminished or even adverse vascular effects, potentially explaining why extremely large HDL particles at very high concentrations are deleterious^42^. Finally, experimental evidence of a biphasic effect of HDL on endothelial progenitor cell angiogenesis provides a biological basis for the non-linear risk observed at the low end of NHHR distribution^43^. Conversely, extremely low non-HDL-C levels may reflect reduced TC levels, potentially indicating malnutrition or frailty, which are associated with a higher risk of adverse outcome^44^. Collectively, these mechanisms provide a biologically plausible explanation for the elevated cardiovascular risk observed in patients with very low NHHR.

When examining the individual components of MACE, the observed U-shaped association of NHHR was primarily driven by all-cause mortality, whereas the highest NHHR quintile was associated with myocardial infarction (MI), and no significant relationship was observed for stroke. The U-shaped relationship with mortality is consistent with many previous studies. Yu et al. reported a U-shaped association between NHHR and mortality in U.S. adults with diabetes^45^, and Zeng et al. identified a similar relationship between non–HDL-C and both all-cause and cardiovascular mortality in men not receiving statins^46^. In addition, several studies have reported that very high HDL-C levels were paradoxically linked to increased mortality^38,47–50^. By contrast, MI—a prototypical atherosclerotic event—showed a more linear association with NHHR. Cui et al. found that higher NHHR was positively associated with angina^51^, and a previous study demonstrated that elevated non–HDL-C predicted a greater risk of non-fatal MI^52^, which is consistent with our findings. For stroke, our study did not identify a significant association. Elsa et al. reported that the causal effect of cumulative LDL-C on stroke was much weaker than that on coronary heart disease^53^, whereas another study observed a positive association between NHHR and stroke in the U.S. general population^54^. Differences in study populations and the influence of competing risks may partly explain the discrepancy between our findings and those reports, the association and mechanisms between NHHR and stroke require further study.

Our subgroup analysis showed that among PAD patients in the lowest NHHR group, those with DM had a significantly higher risk of MACE compared with their non-diabetic peers. This finding is consistent with previous studies reporting that NHHR is a stronger predictor of cardiovascular outcomes in patients with type 2 diabetes^55^. We hypothesize that poor nutrition or the plaque-promoting effects of excessively high HDL-C^39^, combined with diabetes-driven vascular injury through insulin resistance, chronic low-grade inflammation, and oxidative stress^44^, may exacerbate the deleterious effects of dysregulated lipid metabolism observed at very low NHHR levels. This multi-hit pathophysiology could amplify vascular injury and thereby contribute to an increased risk of MACE. The mechanistic interactions between these pathways warrant further investigation.

As modifiable predictors of MACE, lipid indicators are influenced by medications and lifestyle changes. Therefore, we examined the association of NHHR trajectories with MACE incidence. The trajectory analysis revealed a trend consistent with our baseline NHHR findings, showing that NHHR above the optimal range were associated with higher MACE risk, with risk increasing progressively as NHHR rises. Liu et al. similarly reported that high cumulative NHHR exposure was associated with a higher risk of MI, indirectly supporting our findings^56^. A possible mechanism is that higher NHHR reflects elevated non-HDL-C, consistently tied to greater cardiovascular risk in prior studies^57^. Although the incidence of MACE in patients with NHHR below the optimal range was not statistically different from the optimal group, a trend towards higher risk was observed. This lack of significance may stem from requiring at least four NHHR measurements for trajectory analysis, excluding many patients, especially those with lower non-HDL-C less frequently tested. Notably, our findings revealed that lowering elevated NHHR to the optimal range was associated with a MACE incidence comparable to that of the optimal group. This observation is indirectly supported by previous studies^36^, which show that achieving and sustaining target non-HDL-C levels post-MI is associated with a reduced MACE risk^58,59^. Collectively, these findings suggest that long-term efforts to optimize NHHR in PAD patients could potentially lower the incidence of MACE.

While our study suggests potential prognostic value of both baseline NHHR and its longitudinal trajectories in PAD patients, several limitations should be acknowledged. First, as an observational study, it establishes associations rather than causation and may be subject to unmeasured confounding factors, including the absence of data on lifestyle factors such as smoking and physical activity, which could influence the stability of our results. Second, the underdiagnosis of PAD in the Hong Kong population and the absence of detailed subtype information may have limited our ability to perform stratified analyses by disease severity. Moreover, given that our study cohort was predominantly drawn from the general Hong Kong population, caution is warranted when extrapolating these findings to other ethnic groups. Third, potential selection bias may exist due to the exclusion of patients without baseline NHHR values and the requirement of ≥ 4 measurements for trajectory analyses. We acknowledge that such bias cannot be completely ruled out. Nonetheless, most baseline characteristics were broadly comparable between included and excluded patients, which suggests that the impact of this bias is likely limited and that our analytic cohorts remain reasonably representative. Further randomized controlled trials and large-scale data analyses are needed to confirm the validity of our observations and explore their applicability across diverse patient populations.

## Conclusion

This study suggests that NHHR is a potential predictor of MACE in patients with PAD. Our analysis revealed a U-shaped relationship between baseline NHHR and MACE risk, with trajectory analysis showing a similar pattern. Regular monitoring of NHHR may enhance MACE risk stratification in PAD patients, and maintaining NHHR within an optimal range may support individualized lipid management to improve long-term outcomes.

## Supplementary Information

Below is the link to the electronic supplementary material.

Supplementary Material 1

## Data Availability

The datasets generated and/or analysed during the current study were provided by the Hong Kong Hospital Authority and are available from the corresponding author on reasonable request.
