# Supplementary material for: Baseline and longitudinal patterns of Non-HDL-C to HDL-C ratio (NHHR) and major adverse cardiovascular events in peripheral artery disease
Source: Sci Rep. 2025 Oct 15;15:35993. doi: 10.1038/s41598-025-19856-0 (PMC12528666; doi:10.1038/s41598-025-19856-0)
Supplement: Supplementary file 1 — Supplementary Material 1 [file 41598_2025_19856_MOESM1_ESM.docx]

**Baseline and Longitudinal Patterns of Non-HDL-C to HDL-C Ratio (NHHR) and Major Adverse Cardiovascular Events in Peripheral Artery Disease**

**Additional File Information**

Figure S1. Flowchart of the Study Participants.

Figure S2. Distribution of Baseline NHHR.

Figure S3. Event Rate of MACE by NHHR Category (Events/N).

Figure S4. Kaplan-Meier Curves for NHHR Categories and MACE.

Figure S5. Association of baseline NHHR with all-cause mortality.

Figure S6. Association of baseline NHHR with non-fatal myocardial infarction.

Figure S7. Association of baseline NHHR with non-fatal stroke.

Figure S8. Trajectory of Mean NHHR Over Follow-Up After Excluding Patients with a Time Interval Between the First and Last NHHR Measurements Less Than 1 Year.

Figure S9. HRs (95%CIs) for MACE Across NHHR Trajectory Groups After Excluding Patients with a Time Interval Between the First and Last NHHR Measurements Less Than 1 Year.

Table S1. ICD-9-CM Codes for Comorbidities and Outcomes.

Table S2. Table S2. Variance Inflation Factors (VIFs) for covariates in the multivariable Cox model.

Table S3. Baseline Characteristics of Patients with and Without Baseline NHHR Measurement.

Table S4. Baseline Characteristics of Patients According to the Number of NHHR Measurements (<4 vs ≥4).

Table S5. HRs (95% CIs) for MACE by Baseline NHHR Quintiles.

Table S6. Incremental predictive value of NHHR beyond LDL-C and non–HDL-C for predicting MACE.

Table S7. Association between baseline NHHR and risk of all cause death.

Table S8. Association between baseline NHHR and risk of non-fatal myocardial infarction.

Table S9. Association between baseline NHHR and risk of non-fatal stroke.

Table S10. Subgroup analysis of the associations between NHHR and MACE.

Table S11. Average Posterior Probability for Each Latent Trajectory Class.

Table S12. HRs (95% CIs) for MACE Excluding Participants from the First 1 Year of Follow-Up.

Table S13. HRs (95% CIs) for MACE Excluding Participants with a History of Stroke or myocardial infarction.

Table S14. HRs (95% CIs) for MACE Excluding Participants with Neoplastic Diseases, Hematologic Disorders, and Advanced Renal or Hepatic Insufficiency.

Table S15. HRs (95% CIs) for MACE Not Excluding Patients with Events in the First 3 Months of Follow-Up.

Table S16. HRs (95% CIs) for MACE in Cox Models with Different Confounders.

**Figure S1. Flowchart of the Study Participants.**


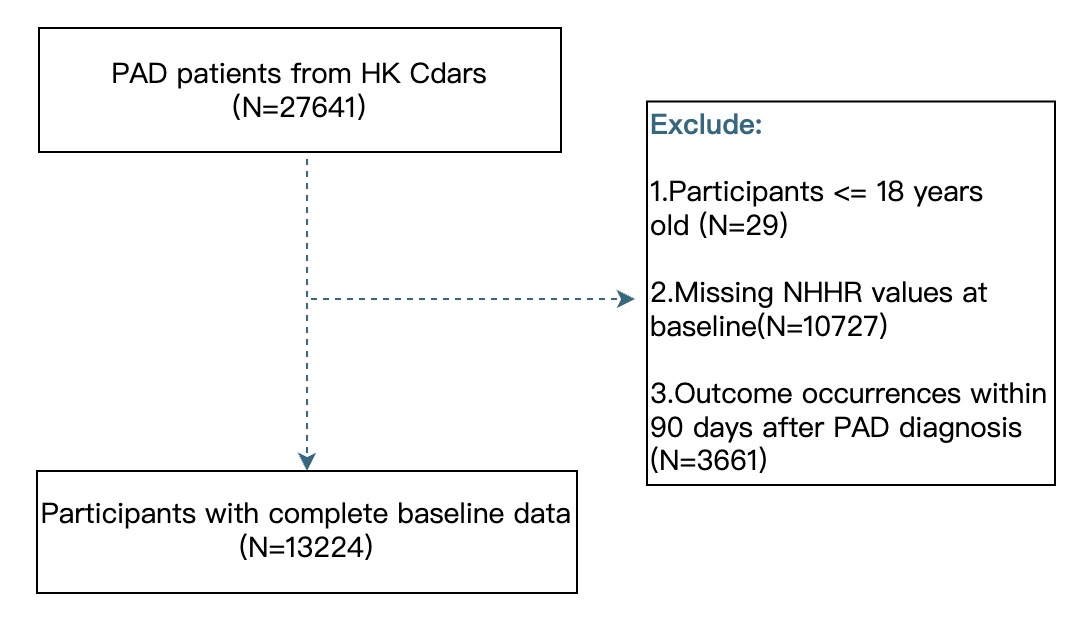


**Figure S2. Distribution of Baseline NHHR.**


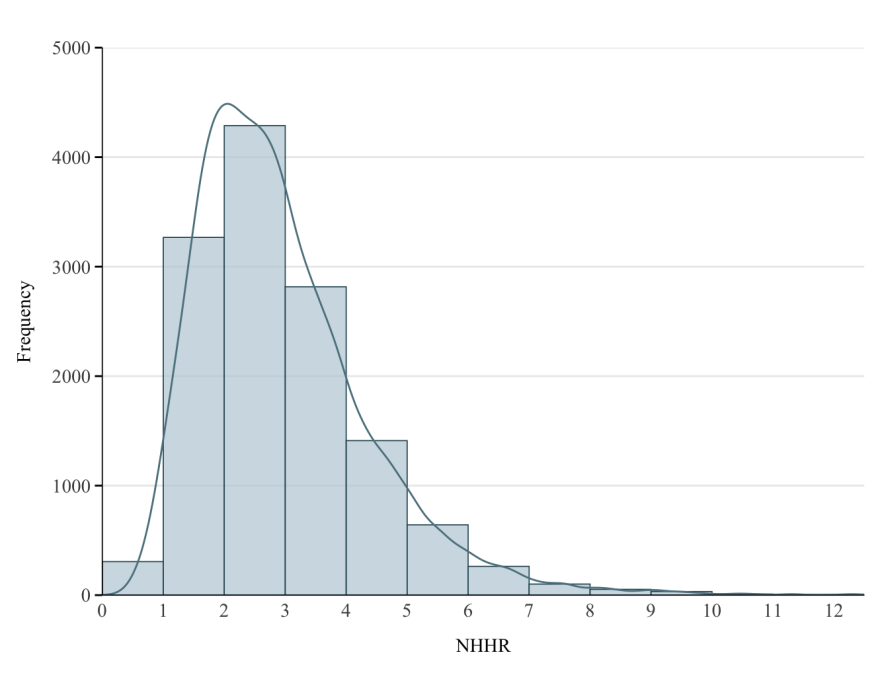


**Figure S3. Event Rate of MACE by NHHR Category (Events/N).**


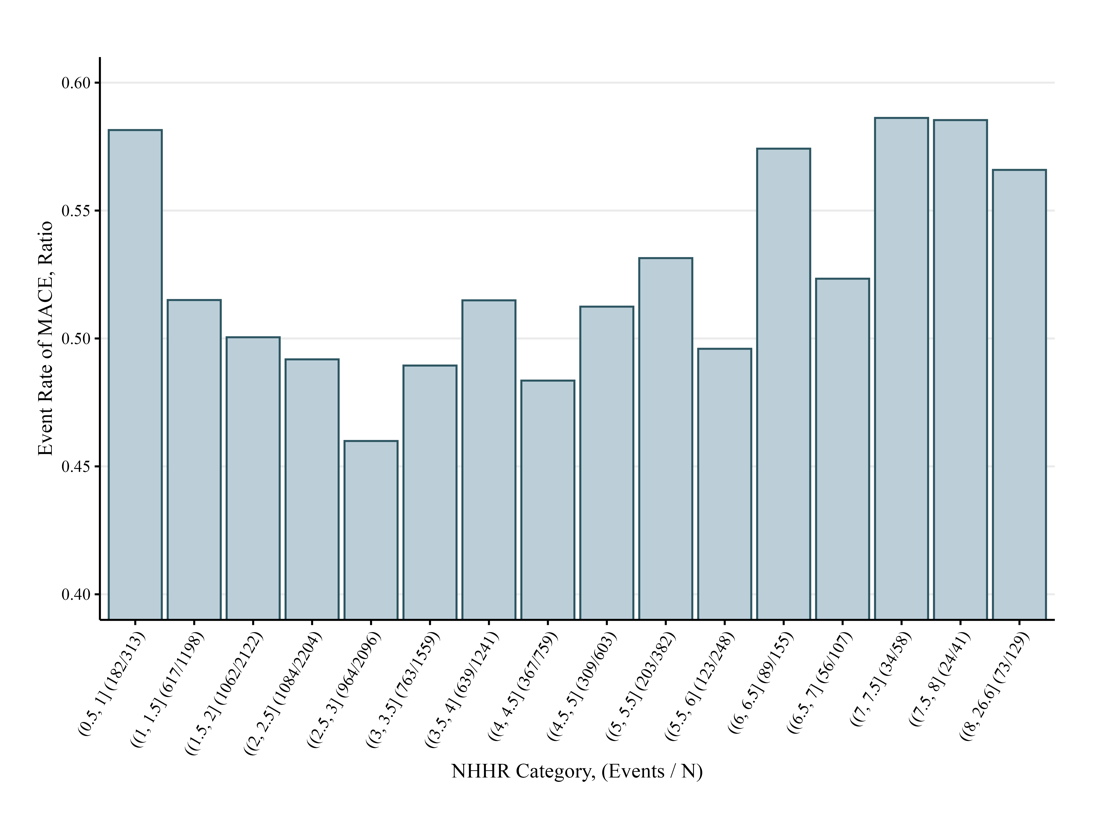


**Figure S4. Kaplan-Meier Curves for NHHR Categories and MACE.**


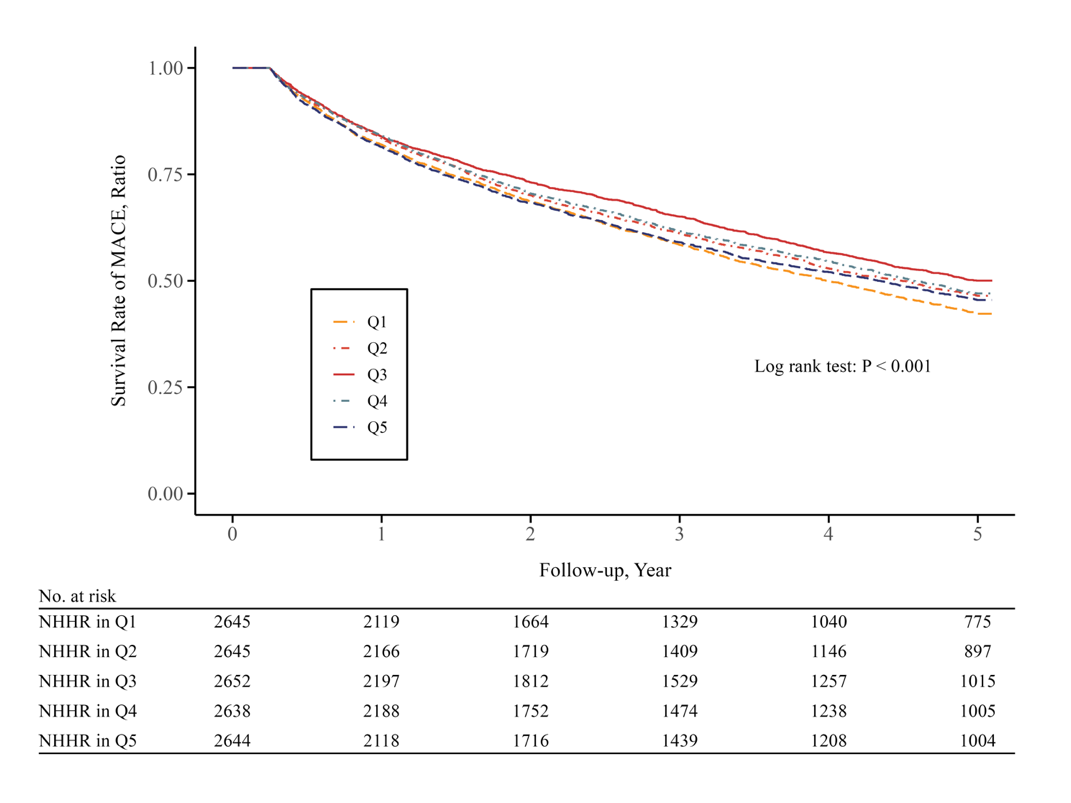


**Note:** NHHR, non–high-density lipoprotein cholesterol to high-density lipoprotein cholesterol ratio; MACE, major adverse cardiovascular events.

**Figure S5. Association of baseline NHHR with all-cause mortality.**

**
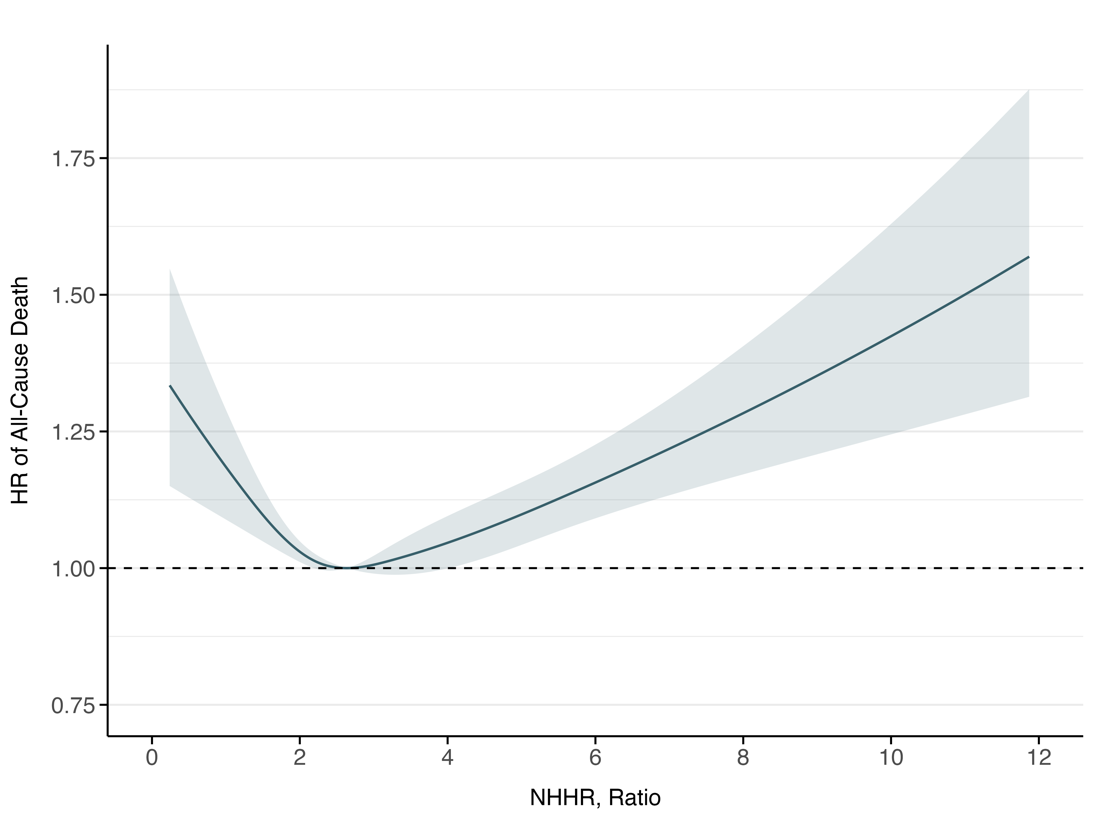
**

**Note:** HR is represented by solid lines, with 95% CIs shown as shaded areas. Model was adjusted for sex, age, hypertension, DM, CAD, stroke, HbA1c, eGFR, and the use of antiplatelets, ACEI/ARBs, and statins. HR hazard ratio, CI confidence interval, DM diabetes mellitus, CAD coronary artery disease, HbA1c glycated hemoglobin, eGFR estimated glomerular filtration rate, ACEI angiotensin-converting enzyme inhibitor, ARBs angiotensin receptor blockers.

**Figure S6. Association of baseline NHHR with non- fatal myocardial infarction.**

**
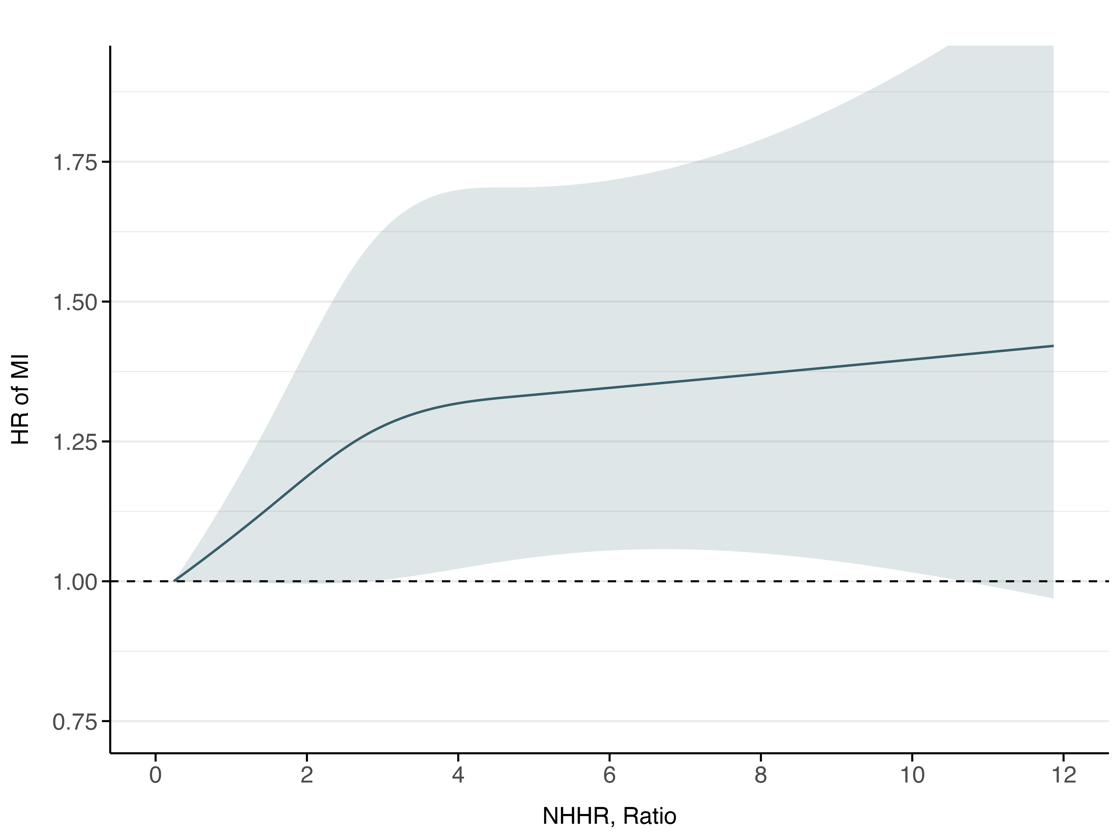
**

**Note:** HR is represented by solid lines, with 95% CIs shown as shaded areas. Model was adjusted for sex, age, hypertension, DM, CAD, stroke, HbA1c, eGFR, and the use of antiplatelets, ACEI/ARBs, and statins. HR hazard ratio, CI confidence interval, DM diabetes mellitus, CAD coronary artery disease, HbA1c glycated hemoglobin, eGFR estimated glomerular filtration rate, ACEI angiotensin-converting enzyme inhibitor, ARBs angiotensin receptor blockers.

**Figure S7. Association of baseline NHHR with non- fatal stroke.**

**
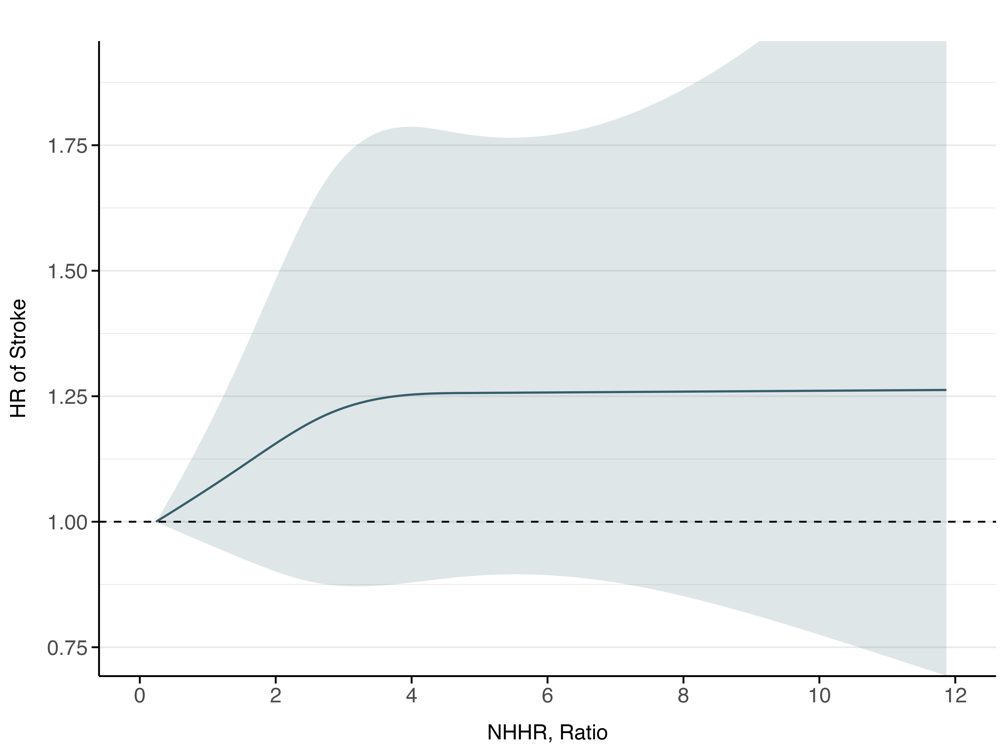
**

**Note:** HR is represented by solid lines, with 95% CIs shown as shaded areas. Model was adjusted for sex, age, hypertension, DM, CAD, stroke, HbA1c, eGFR, and the use of antiplatelets, ACEI/ARBs, and statins. HR hazard ratio, CI confidence interval, DM diabetes mellitus, CAD coronary artery disease, HbA1c glycated hemoglobin, eGFR estimated glomerular filtration rate, ACEI angiotensin-converting enzyme inhibitor, ARBs angiotensin receptor blockers.

**Figure S8. Trajectory of Mean NHHR Over Follow-Up After Excluding Patients with a Time Interval Between the First and Last NHHR Measurements Less Than 1 Year.**


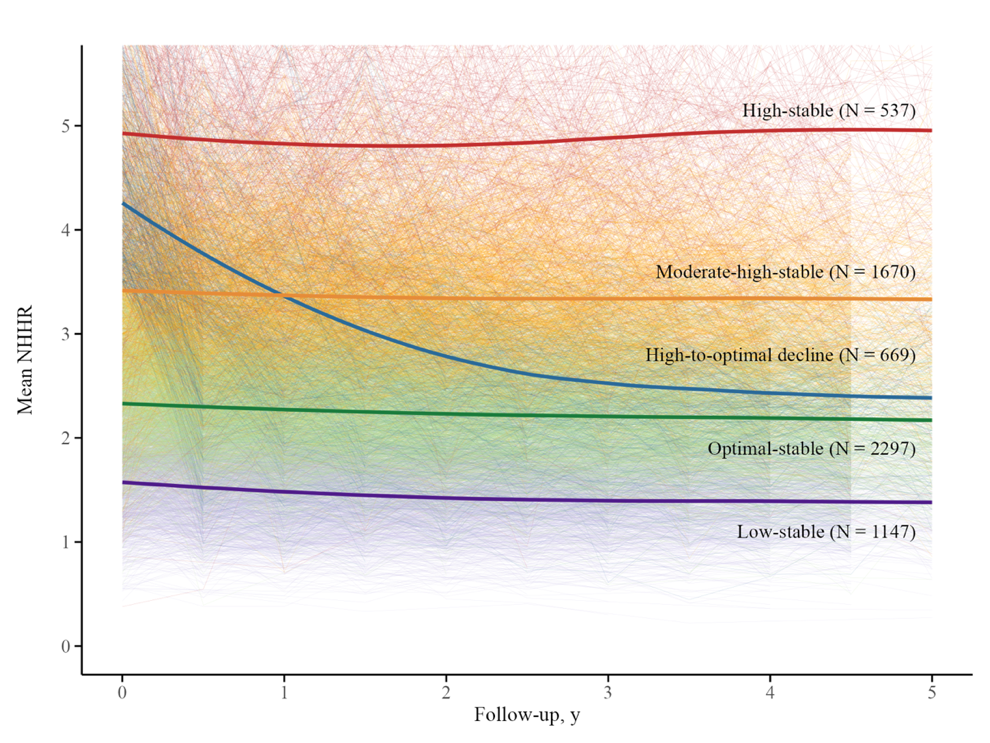


**Figure S9. HRs (95%CIs) for MACE Across NHHR Trajectory Groups After Excluding Patients with a Time Interval Between the First and Last NHHR Measurements Less Than 1 Year.**


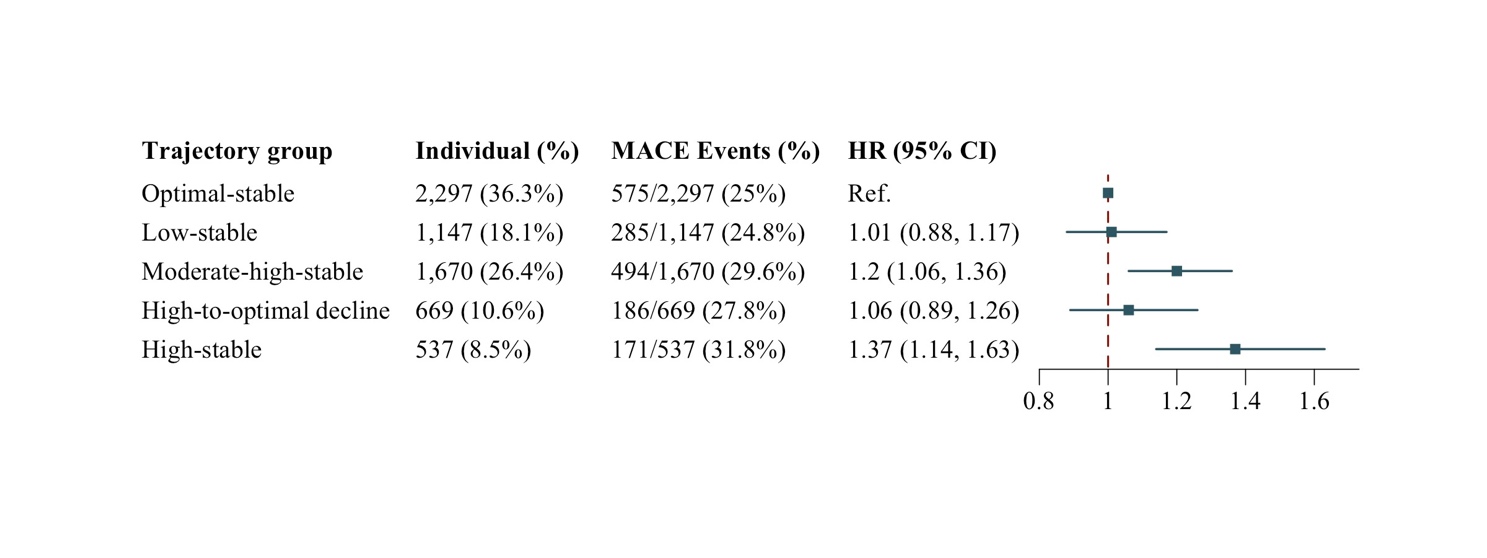


**Note:** Model was adjusted for sex, age, hypertension, DM, CAD, stroke, HbA1c, eGFR, and the use of antiplatelets, ACEI/ARBs, and statins. HR hazard ratio, CI confidence interval, DM diabetes mellitus, CAD coronary artery disease, HbA1c glycated hemoglobin, eGFR estimated glomerular filtration rate, ACEI angiotensin-converting enzyme inhibitor, ARBs angiotensin receptor blockers.

**Table S1. ICD-9-CM Codes for Comorbidities and Outcomes.**

| **Disease** | **ICD-9-CM** |
| --- | --- |
| PAD | 440.20, 440.21, 440.22, 440.23, 440.24, 440.29, 440.4, 440.8, 440.9, 443, 443.1, 443.2, 443.8, 443.81, 443.82, 443.89, and 443.9. |
| Hypertension | 401, 402, 405, 403, 404, 402.91, 404.91, 404.93 |
| Coronary artery disease | 410, 411, 412, 413, 414, 429.79 |
| Chronic kidney disease | 250.4, 403, 404, 581, 582, 583, 585, 586, 588, 402.91, 404.91, 404.93 |
| Dyslipidemia | 272.0, 272.1, 272.2, 272.3, 272.4 |
| Diabetes | 250.00 |
| Congestive heart failure | 398.91, 402.91, 404.91, 404.9, ,425, 428 |
| Dysrhythmia | 426, 427.0, 427.1, 427.2, 427.4, 427.6, 427.8, 427.9 |
| MI | 410 |
| Stroke | 433, 434, 435, 436, 437, 438, 430, 431, 432 |
| Non-fatal stroke(outcome) | 433, 434 |

**Table S2. Variance Inflation Factors (VIFs) for covariates in the multivariable Cox model.**

| **Covariates** | **VIF** |
| --- | --- |
| Sex (male) | 1.096 |
| Age | 1.238 |
| Hypertension | 1.224 |
| DM | 1.268 |
| CAD | 1.191 |
| Stroke | 1.077 |
| eGFR | 1.186 |
| HbA1c | 1.087 |
| Antiplatelet therapy | 1.183 |
| Statin therapy | 1.220 |
| ACEI/ARBs therapy | 1.151 |

**Note:** VIF variance inflation factor, DM diabetes mellitus, CAD coronary artery disease, eGFR, estimated glomerular filtration rate, HbA1c, hemoglobin A1c, ACEI/ ARBs angiotensin-converting enzyme inhibitor/ angiotensin II receptor blockers.

**Table S3. Baseline Characteristics of Patients with and without Baseline NHHR Measurement.**

| Parameters | Without baseline NHHR  N=14,417 | With baseline NHHR  N = 13,224 | P |
| --- | --- | --- | --- |
| NHHR | 2.78 (1.99, 3.93) | 2.74 (1.96, 3.72) | 0.017 |
| Demographic |  |  |  |
| Age, years | 80.00 (71.00, 87.00) | 74.00 (65.00, 82.00) | <0.001 |
| Sex (Male), % | 7189 (49.9%) | 8035 (60.8%) | <0.001 |
| Comorbidities |  |  |  |
| Hypertension, % | 7309 (50.7%) | 7700 (58.2%) | <0.001 |
| CAD, % | 3761 (26.1%) | 3927 (29.7%) | <0.001 |
| Stroke, % | 4233 (29.4%) | 3021 (22.8%) | <0.001 |
| Dyslipidemia, % | 1147 (8.0%) | 876 (6.6%) | <0.001 |
| CKD, % | 2398 (16.6%) | 2896 (21.9%) | <0.001 |
| Diabetes, % | 5434 (37.7%) | 7160 (54.1%) | <0.001 |
| Laboratory |  |  |  |
| LDL-C, mmol/L | 2.36 (1.77, 3.08) | 2.32 (1.78, 3.01) | 0.098 |
| TC, mmol/L | 4.05 (3.32, 4.85) | 4.20 (3.50, 5.00) | <0.001 |
| HDL-C, mmol/L | 1.03 (0.82, 1.30) | 1.11 (0.91, 1.36) | <0.001 |
| TG, mmol/L | 1.18 (0.90, 1.62) | 1.27 (0.92, 1.78) | <0.001 |
| eGFR, mL/min/1.73 m² | 52.47 (27.57, 77.18) | 58.20 (36.81, 80.08) | <0.001 |
| HbA1c, % | 6.80 (5.90, 8.20) | 7.10 (6.20, 8.48) | <0.001 |
| Medication |  |  |  |
| Anticoagulant, % | 1013 (7.0%) | 1073 (8.1%) | <0.001 |
| ACEI/ARBs, % | 5313 (36.9%) | 7169 (54.2%) | <0.001 |
| β-blockers, % | 4240 (29.4%) | 5093 (38.5%) | <0.001 |
| Statin, % | 3943 (27.3%) | 7675 (58.0%) | <0.001 |
| Antiplatelet, % | 8566 (59.4%) | 9485 (71.7%) | <0.001 |
| Antihyperglycemic  treatment, % | 2775 (19.2%) | 4058 (30.7%) | <0.001 |

**Note:** Values are presented as mean ± SD, median (IQR), or n (%), as appropriate.

NHHR non–high-density lipoprotein cholesterol to high-density lipoprotein cholesterol ratio, CAD coronary artery disease, CKD chronic kidney disease, TC total cholesterol, LDL-C low-density lipoprotein cholesterol, HDL-C high-density lipoprotein cholesterol, TG triglycerides, eGFR estimated glomerular filtration rate, HbA1c glycated hemoglobin, ACEI angiotensin-converting enzyme inhibitor, ARBs angiotensin receptor blockers.

**Table S4. Baseline Characteristics of Patients According to the Number of NHHR Measurements (<4 vs ≥4).**

| Parameters | NHHR measurement<4  N=6,667 | NHHR measurement>=4  N = 6,557 | P |
| --- | --- | --- | --- |
| NHHR | 2.68 (1.92, 3.67) | 2.68 (1.95, 3.65) | 0.415 |
| Demographic |  |  |  |
| Age, years | 77.00 (68.00, 84.00) | 71.00 (63.00, 79.00) | <0.001 |
| Sex (Male), % | 3882 (58.2%) | 4153 (63.3%) | <0.001 |
| Comorbidities |  |  |  |
| Hypertension, % | 3995 (59.9%) | 3705 (56.5%) | <0.001 |
| CAD, % | 1991 (29.9%) | 1936 (29.5%) | 0.685 |
| Stroke, % | 1741 (26.1%) | 1280 (19.5%) | <0.001 |
| Dyslipidemia, % | 492 (7.4%) | 384 (5.9%) | <0.001 |
| CKD, % | 1563 (23.4%) | 1333 (20.3%) | <0.001 |
| Diabetes, % | 3318 (49.8%) | 3842 (58.6%) | <0.001 |
| Laboratory |  |  |  |
| LDL-C, mmol/L | 2.38 (1.80, 3.07) | 2.36 (1.84, 3.04) | 0.729 |
| TC, mmol/L | 4.22 (3.53, 5.03) | 4.21 (3.60, 5.00) | 0.347 |
| HDL-C, mmol/L | 1.13 (0.92, 1.40) | 1.13 (0.93, 1.38) | 0.657 |
| TG, mmol/L | 1.20 (0.90, 1.69) | 1.30 (0.94, 1.84) | <0.001 |
| eGFR, mL/min/1.73 m² | 60.12 (35.41, 84.12) | 67.02 (44.40, 86.88) | <0.001 |
| HbA1c, % | 6.80 (6.00, 8.20) | 7.28 (6.40, 8.64) | <0.001 |
| Medication |  |  |  |
| Anticoagulant, % | 578 (8.7%) | 495 (7.5%) | 0.020 |
| ACEI/ARBs, % | 3315 (49.7%) | 3854 (58.8%) | <0.001 |
| β-blockers, % | 2470 (37.0%) | 2623 (40.0%) | <0.001 |
| Statin, % | 3329 (49.9%) | 4346 (66.3%) | <0.001 |
| Antiplatelet, % | 4743 (71.1%) | 4742 (72.3%) | 0.138 |
| Antihyperglycemic  treatment, % | 1881 (28.2%) | 2177 (33.2%) | <0.001 |

**Note:** Values are presented as mean ± SD, median (IQR), or n (%), as appropriate.

NHHR non–high-density lipoprotein cholesterol to high-density lipoprotein cholesterol ratio, CAD coronary artery disease, CKD chronic kidney disease, TC total cholesterol, LDL-C low-density lipoprotein cholesterol, HDL-C high-density lipoprotein cholesterol, TG triglycerides, eGFR estimated glomerular filtration rate, HbA1c glycated hemoglobin, ACEI angiotensin-converting enzyme inhibitor, ARBs angiotensin receptor blockers.

**Table S5. HRs (95% CIs) for MACE by Baseline NHHR Quintiles.**

|  | NHHR Q1 group  (N = 2,654) | | NHHR Q2 group  (N = 2,645) | | NHHR Q3 group  (N = 2,652) | NHHR Q4 group  (N = 2,638) | | NHHR Q5 group  (N = 2,644) | |
| --- | --- | --- | --- | --- | --- | --- | --- | --- | --- |
| Model | HR (95% CI) | P value | HR (95% CI) | P value | Reference | HR (95% CI) | P value | HR (95% CI) | P value |
| Unadjusted | 1.21 (1.12, 1.31) | <0.001 | 1.11 (1.03, 1.2) | 0.008 | 1.00 | 1.08 (1, 1.17) | 0.048 | 1.15 (1.07, 1.25) | <0.001 |
| Adjusted* | 1.17 (1.08, 1.27) | <0.001 | 1.09 (1.01, 1.18) | 0.027 | 1.00 | 1.08 (1, 1.17) | 0.047 | 1.16 (1.07, 1.25) | <0.001 |

**Note:** The model was adjusted for sex, age, hypertension, DM, CAD, stroke, HbA1c, eGFR, and the use of antiplatelets, ACEI/ARBs, statins. HR hazard ratio, CI confidence interval, DM diabetes mellitus, CAD coronary artery disease,

HbA1c glycated hemoglobin, eGFR estimated glomerular filtration rate, ACEI angiotensin-converting enzyme inhibitor,

ARBs angiotensin receptor blockers.

**Table S6. Incremental predictive value of NHHR beyond LDL-C and non–HDL-C for predicting MACE.**

| **Model** | **C-index** | **95% CI** | **IDI** | **95% CI** | **NRI** | **95% CI** | **Likelihood Ratio Test** |
| --- | --- | --- | --- | --- | --- | --- | --- |
| Confounders + LDL-C | 0.6287 | (0.6206, 0.6369) | Ref |  | Ref |  |  |
| Confounders + LDL-C + NHHR | 0.6821 | (0.6756, 0.6886) | 0.000797 | (0.000294, 0.001301) | 0.065 | (0.0316, 0.0984) | P<0.001 |
| Confounders + non-HDL-C | 0.6822 | (0.6763, 0.6892) | Ref |  | Ref |  |  |
| Confounders + non-HDL-C + NHHR | 0.6832 | (0.6767, 0.6897) | 0.000899 | (0.000325, 0.001472) | 0.0497 | (0.01559, 0.0838) | P<0.001 |

**Note:** IDI and NRI were estimated by incremental analysis with and without NHHR. Likelihood ratio test P values indicate the improvement in model fit after adding NHHR. Ref indicates the reference model (without NHHR). NHHR non–high-density lipoprotein cholesterol to high-density lipoprotein cholesterol ratio, LDL-C low-density lipoprotein cholesterol, non-HDL-C non-high-density lipoprotein cholesterol, IDI integrated discrimination improvement, NRI net reclassification improvement, CI confidence interval.

**Table S7. Association between baseline NHHR and risk of all cause death.**

|  | | NHHR Q1 group  (N = 2,645) | | NHHR Q2 group  (N = 2,645) | | | NHHR Q3 group  (N = 2,652) | NHHR Q4 group  (N = 2,638) | | | NHHR Q5 group  (N = 2,644) | | |  |
| --- | --- | --- | --- | --- | --- | --- | --- | --- | --- | --- | --- | --- | --- | --- |
| Model | HR (95% CI) | | P value | | HR (95% CI) | P value | Reference | | HR (95% CI) | P value | | HR (95% CI) | P value | |
| Unadjusted | 1.18 (0.98, 1.43) | | 0.078 | | 1.01 (0.83, 1.23) | 0.903 | 1.00 | | 1.12 (0.93, 1.35) | 0.228 | | 1.1 (0.91, 1.33) | 0.326 | |
| Adjusted* | 1.21 (1.11, 1.31) | | <0.001 | | 1.08 (0.99, 1.18) | 0.065 | 1.00 | | 1.05 (0.96, 1.14) | 0.277 | | 1.14 (1.05, 1.24) | 0.002 | |

**Note:** The model was adjusted for sex, age, hypertension, DM, CAD, stroke, HbA1c, eGFR, and the use of antiplatelets, ACEI/ARBs, statins. HR hazard ratio, CI confidence interval, DM diabetes mellitus, CAD coronary artery disease, HbA1c glycated hemoglobin, eGFR estimated glomerular filtration rate, ACEI angiotensin-converting enzyme inhibitor, ARBs angiotensin receptor blockers.

**Table S8. Association between baseline NHHR and risk of non-fatal myocardial infarction.**

|  | NHHR Q1 group  (N = 2,645) | | NHHR Q2 group  (N = 2,645) | | NHHR Q3 group  (N = 2,652) | | NHHR Q4 group  (N = 2,638) | | NHHR Q5 group  (N = 2,644) | |
| --- | --- | --- | --- | --- | --- | --- | --- | --- | --- | --- |
| Model | | Reference | HR (95% CI) | P value | HR (95% CI) | P value | HR (95% CI) | P value | HR (95% CI) | P value |
| Unadjusted | | 1.00 | 1.13 (0.96, 1.34) | 0.142 | 1.01 (0.86, 1.2) | 0.881 | 1.15 (0.98, 1.36) | 0.086 | 1.29 (1.1, 1.52) | 0.002 |
| Adjusted* | | 1.00 | 1.13 (0.96, 1.34) | 0.138 | 1.05 (0.88, 1.24) | 0.606 | 1.18 (1, 1.39) | 0.056 | 1.27 (1.08, 1.49) | 0.005 |

**Note:** The model was adjusted for sex, age, hypertension, DM, CAD, stroke, HbA1c, eGFR, and the use of antiplatelets, ACEI/ARBs, statins. HR hazard ratio, CI confidence interval, DM diabetes mellitus, CAD coronary artery disease, HbA1c glycated hemoglobin, eGFR estimated glomerular filtration rate, ACEI angiotensin-converting enzyme inhibitor, ARBs angiotensin receptor blockers.

**Table S9. Association between baseline NHHR and risk of non-fatal stroke.**

|  | NHHR Q1 group  (N = 2,645) | | NHHR Q2 group  (N = 2,645) | | NHHR Q3 group  (N = 2,652) | | NHHR Q4 group  (N = 2,638) | | NHHR Q5 group  (N = 2,644) | |
| --- | --- | --- | --- | --- | --- | --- | --- | --- | --- | --- |
| Model | | Reference | HR (95% CI) | P value | HR (95% CI) | P value | HR (95% CI) | P value | HR (95% CI) | P value |
| Unadjusted | | 1.00 | 0.88 (0.7, 1.11) | 0.276 | 0.96 (0.77, 1.2) | 0.729 | 1.05 (0.84, 1.3) | 0.682 | 1.01 (0.81, 1.26) | 0.911 |
| Adjusted* | | 1.00 | 0.91 (0.73, 1.15) | 0.439 | 1.01 (0.81, 1.27) | 0.916 | 1.12 (0.89, 1.39) | 0.334 | 1.12 (0.89, 1.4) | 0.348 |

**Note:** The model was adjusted for sex, age, hypertension, DM, CAD, stroke, HbA1c, eGFR, and the use of antiplatelets, ACEI/ARBs, statins. HR hazard ratio, CI confidence interval, DM diabetes mellitus, CAD coronary artery disease, HbA1c glycated hemoglobin, eGFR estimated glomerular filtration rate, ACEI angiotensin-converting enzyme inhibitor, ARBs angiotensin receptor blockers.

**Table S10. Subgroup analysis of the associations between NHHR and MACE.**

| **Subgroup** | **NHHR Q1 group**  **(N = 2,654)**  **HR (95% CI),**  **P Value** | **NHHR Q2 group**  **(N = 2,645)**  **HR (95% CI),**  **P Value** | **NHHR Q3 group**  **(N = 2,652)**  **(Reference)** | **NHHR Q4 group**  **(N = 2,638)**  **HR (95% CI),**  **P Value** | **NHHR Q5 group**  **(N = 2,644)**  **HR (95% CI),**  **P Value** |
| --- | --- | --- | --- | --- | --- |
| Male | 1.13 (1.01, 1.25),  P = 0.026 | 1.04 (0.94, 1.16),  P = 0.408 | Ref. | 1.07 (0.97, 1.19),  P = 0.177 | 1.09 (0.98, 1.20),  P = 0.110 |
| Female | 1.23 (1.09, 1.39),  P < 0.001 | 1.17 (1.04, 1.32),  P = 0.011 | Ref. | 1.10 (0.97, 1.25),  P = 0.121 | 1.29 (1.14, 1.46),  P < 0.001 |
| Age < 65 | 0.99 (0.81, 1.22),  P = 0.944 | 1.00 (0.81, 1.22),  P = 0.966 | Ref. | 0.97 (0.80, 1.18),  P = 0.744 | 1.07 (0.90, 1.29),  P = 0.431 |
| Age >= 65 | 1.23 (1.13, 1.34),  P < 0.001 | 1.13 (1.03, 1.23),  P = 0.006 | Ref. | 1.08 (0.99, 1.18),  P = 0.067 | 1.10 (1.01, 1.20),  P = 0.035 |
| hypertension | 1.12 (0.98, 1.28),  P = 0.084 | 1.10 (0.96, 1.26),  P = 0.158 | Ref. | 1.04 (0.92, 1.19),  P = 0.527 | 1.21 (1.06, 1.38),  P = 0.004 |
| Non-hypertension | 1.19 (1.08, 1.32),  P < 0.001 | 1.09 (0.99, 1.20),  P = 0.083 | Ref. | 1.11 (1.01, 1.23),  P = 0.032 | 1.13 (1.02, 1.25),  P = 0.014 |
| diabetes | 1.27 (1.13, 1.44),  P < 0.001 | 1.10 (0.97, 1.24),  P = 0.147 | Ref. | 1.09 (0.96, 1.23),  P = 0.196 | 1.15 (1.01, 1.31),  P = 0.033 |
| Non-diabetes | 1.09 (0.98, 1.20),  P = 0.122 | 1.09 (0.99, 1.21),  P = 0.095 | Ref. | 1.08 (0.98, 1.20),  P = 0.113 | 1.16 (1.05, 1.28),  P = 0.003 |
| CAD | 1.17 (1.06, 1.29),  P = 0.002 | 1.09 (0.99, 1.20),  P = 0.086 | Ref. | 1.09 (0.99, 1.21),  P = 0.075 | 1.15 (1.04, 1.27),  P = 0.005 |
| Non- CAD | 1.18 (1.04, 1.34),  P = 0.012 | 1.09 (0.96, 1.25),  P = 0.175 | Ref. | 1.07 (0.94, 1.23),  P = 0.287 | 1.18 (1.04, 1.35),  P = 0.013 |

**Table S11. Average Posterior Probability for Each Latent Trajectory Class.**

|  | **Optimal** | **Low** | **Moderate High** | **High decreasing** | **High** |
| --- | --- | --- | --- | --- | --- |
| Average posterior probability | 0.915 | 0.932 | 0.918 | 0.922 | 0.931 |

**Table S12. HRs (95% CIs) for MACE Excluding Participants from the First 1 Year of Follow-Up.**

|  | NHHR Q1 group  (N = 2,244) | | NHHR Q2 group  (N = 2,247) | | NHHR Q3 group  (N = 2,241) | NHHR Q4 group  (N = 2,244) | | NHHR Q5 group  (N = 2,244) | |
| --- | --- | --- | --- | --- | --- | --- | --- | --- | --- |
| Model | HR (95% CI) | P value | HR (95% CI) | P value | Reference | HR (95% CI) | P value | HR (95% CI) | P value |
| Unadjusted | 1.29 (1.17, 1.41) | <0.001 | 1.16 (1.06, 1.27) | 0.001 | 1.00 | 1.16 (1.05, 1.27) | 0.002 | 1.18 (1.08, 1.29) | <0.001 |
| Adjusted* | 1.25 (1.14, 1.37) | <0.001 | 1.14 (1.04, 1.25) | 0.005 | 1.00 | 1.16 (1.06, 1.28) | 0.001 | 1.2 (1.09, 1.31) | <0.001 |

**Note:** The model was adjusted for sex, age, hypertension, DM, CAD, stroke, HbA1c, eGFR, and the use of antiplatelets, ACEI/ARBs, statins. HR hazard ratio, CI confidence interval, DM diabetes mellitus, CAD coronary artery disease, HbA1c glycated hemoglobin, eGFR estimated glomerular filtration rate, ACEI angiotensin-converting enzyme inhibitor, ARBs angiotensin receptor blockers.

**Table S13. HRs (95% CIs) for MACE Excluding Participants with a History of Stroke or myocardial infarction.**

|  | | NHHR Q1 group  (N = 1,833) | | NHHR Q2 group  (N = 1,836) | | NHHR Q3 group  (N = 1,830) | | | NHHR Q4 group  (N = 1,842) | | | NHHR Q5 group  (N = 1,842) | | |
| --- | --- | --- | --- | --- | --- | --- | --- | --- | --- | --- | --- | --- | --- | --- |
| Model | HR (95% CI) | | P value | HR (95% CI) | P value | | Reference | HR (95% CI) | | P value | HR (95% CI) | | P value |  |
| Unadjusted | 1.21 (1.1, 1.33) | | <0.001 | 1.16 (1.05, 1.28) | 0.003 | | 1.00 | 1.09 (0.99, 1.2) | | 0.092 | 1.18 (1.07, 1.3) | | 0.001 |  |
| Adjusted* | 1.21 (1.1, 1.34) | | <0.001 | 1.16 (1.05, 1.28) | 0.004 | | 1.00 | 1.09 (0.98, 1.2) | | 0.103 | 1.16 (1.05, 1.28) | | 0.004 |  |

**Note:** The model was adjusted for sex, age, hypertension, DM, CAD, stroke, HbA1c, eGFR, and the use of antiplatelets, ACEI/ARBs, statins. HR hazard ratio, CI confidence interval, DM diabetes mellitus, CAD coronary artery disease, HbA1c glycated hemoglobin, eGFR estimated glomerular filtration rate, ACEI angiotensin-converting enzyme inhibitor, ARBs angiotensin receptor blockers.

**Table S14. HRs (95% CIs) for MACE Excluding Participants with Neoplastic Diseases, Hematologic Disorders, and Advanced Renal or Hepatic Insufficiency.**

|  | | **NHHR Q1 group**  **(N = 2,466)** | | **NHHR Q2 group**  **(N = 2,471)** | | | **NHHR Q3 group**  **(N = 2,463)** | | | **NHHR Q4 group**  **(N = 2,469)** | | **NHHR Q5 group**  **(N = 2,469)** | |  |
| --- | --- | --- | --- | --- | --- | --- | --- | --- | --- | --- | --- | --- | --- | --- |
| Model | HR (95% CI) | | P value | | HR (95% CI) | P value | | Reference | HR (95% CI) | | P value | HR (95% CI) | P value | |
| Unadjusted | 1.23 (1.13, 1.33) | | <0.001 | | 1.12 (1.03, 1.21) | 0.007 | | 1.00 | 1.09 (1.01, 1.19) | | 0.029 | 1.17 (1.08, 1.27) | <0.001 | |
| Adjusted* | 1.18 (1.09, 1.28) | | <0.001 | | 1.08 (1, 1.17) | 0.06 | | 1.00 | 1.09 (1, 1.18) | | 0.048 | 1.17 (1.08, 1.27) | <0.001 | |

**Note:** The model was adjusted for sex, age, hypertension, DM, CAD, stroke, HbA1c, eGFR, and the use of antiplatelets, ACEI/ARBs, statins. HR hazard ratio, CI confidence interval, DM diabetes mellitus, CAD coronary artery disease, HbA1c glycated hemoglobin, eGFR estimated glomerular filtration rate, ACEI angiotensin-converting enzyme inhibitor, ARBs angiotensin receptor blockers.

**Table S15. HRs (95% CIs) for MACE Not Excluding Patients with Events in the First 3 Months of Follow-Up.**

|  | | **NHHR Q1 group**  **(N = 3,021)** | | **NHHR Q2 group**  **(N = 3,023)** | | | **NHHR Q3 group**  **(N = 3,020)** | **NHHR Q4 group**  **(N = 3,022)** | | | **NHHR Q5 group**  **(N = 3,022)** | | |  |
| --- | --- | --- | --- | --- | --- | --- | --- | --- | --- | --- | --- | --- | --- | --- |
| Model | HR (95% CI) | | P value | | HR (95% CI) | P value | Reference | | HR (95% CI) | P value | | HR (95% CI) | P value | |
| Unadjusted | 1.2 (1.13, 1.29) | | <0.001 | | 1.07 (1, 1.14) | 0.062 | 1.00 | | 1.06 (0.99, 1.13) | 0.117 | | 1.15 (1.08, 1.24) | <0.001 | |
| Adjusted* | 1.19 (1.1, 1.29) | | <0.001 | | 1.09 (1.01, 1.18) | 0.027 | 1.00 | | 1.09 (1.01, 1.18) | 0.029 | | 1.17 (1.08, 1.26) | <0.001 | |

**Note:** The model was adjusted for sex, age, hypertension, DM, CAD, stroke, HbA1c, eGFR, and the use of antiplatelets, ACEI/ARBs, statins. HR hazard ratio, CI confidence interval, DM diabetes mellitus, CAD coronary artery disease, HbA1c glycated hemoglobin, eGFR estimated glomerular filtration rate, ACEI angiotensin-converting enzyme inhibitor, ARBs angiotensin receptor blockers.

**Table S16. HRs (95% CIs) for MACE in Cox Models with Different Confounders.**

|  | **NHHR Q1 group**  **(N = 2,645)** | | **NHHR Q2 group**  **(N = 2,645)** | | **NHHR Q3 group**  **(N = 2,652)** | **NHHR Q4 group**  **(N = 2,638)** | | **NHHR Q5 group**  **(N = 2,644)** | |
| --- | --- | --- | --- | --- | --- | --- | --- | --- | --- |
| Model | HR (95% CI) | P value | HR (95% CI) | P value | Reference | HR (95% CI) | P value | HR (95% CI) | P value |
| unadjusted | 1.21 (1.12, 1.31) | <0.001 | 1.11 (1.03, 1.2) | 0.008 | 1.00 | 1.08 (1, 1.17) | 0.048 | 1.15 (1.07, 1.25) | <0.001 |
| adjusted | 1.12 (1.03, 1.21) | 0.007 | 1.07 (0.98, 1.15) | 0.115 | 1.00 | 1.14 (1.05, 1.24) | 0.001 | 1.38 (1.27, 1.5) | <0.001 |

**Note:** The model was adjusted for sex, age, hypertension, DM, CAD, stroke, HbA1c, eGFR, TG, and the use of antiplatelets, ACEI/ARBs, statins. HR hazard ratio, CI confidence interval, DM diabetes mellitus, CAD coronary artery disease, HbA1c glycated hemoglobin, eGFR estimated glomerular filtration rate, TG triglycerides, ACEI angiotensin-converting enzyme inhibitor, ARBs angiotensin receptor blockers.
